# Squamous Tissue Lymphocytes in the Esophagus of Controls and Patients with Reflux Esophagitis and Barrett’s Esophagus Are Characterized by a Non-Inflammatory Phenotype

**DOI:** 10.1371/journal.pone.0106261

**Published:** 2014-08-29

**Authors:** Alexandra Lind, Leo Koenderman, Johannes G. Kusters, Peter D. Siersema

**Affiliations:** 1 Department of Respiratory Medicine, University Medical Center Utrecht, Utrecht, the Netherlands; 2 Department of Gastroenterology and Hepatology, University Medical Center Utrecht, Utrecht, the Netherlands; 3 Department of Medical Microbiology, University Medical Center Utrecht, Utrecht, the Netherlands; Cincinnati Children’s Hospital Medical Center, University of Cincinnati College of Medicine, United States of America

## Abstract

**Background and Objective:**

Reflux esophagitis (RE) is characterized by inflammation of the squamous epithelium (SQ) of the esophagus and may progress to Barrett’s esophagus (BE) characterized by intestinal metaplasia. The role of inflammation in this transition has been postulated but lacks experimental evidence. Here, the inflammatory responses in the esophagus of these patients were investigated.

**Patients and Methods:**

Fifty-one esophageal biopsies from with patients BE (n = 19), RE (n = 8) and controls (n = 23) were analyzed. T-cells were analyzed before and after *ex vivo* expansion (14 days) by multicolor flow cytometric analysis. The following markers were studied: CD3, CD4, CD8 (T-cell markers), Granzyme B (marker of cytotoxicity), CD103 (αE/epithelial integrin) and NKg2a (inhibitory receptor on T-cells and NK-cells).

**Results:**

Analysis of *ex vivo* cultures from normal looking SQ from controls, RE patients, and BE patients revealed no significant differences in the number and phenotypes of T-cells. In contrast, tissue from RE was different to normal SQ in four aspects: 1) higher percentages of CD3^+^CD4^+^-cells (72±7% vs 48±6%, p = 0.01) and 2) CD8^+^GranzymeB^+^ -cells (53±11% vs 26±4%, p<0.05), while 3) lower percentages of CD4^+^CD103^+^-cells (45±19% vs 80±3%, p = 0.02) and 4) CD8^+^NKg2a^+^- cells (31±12% vs 44±5%).

**Conclusion:**

Despite the fact that both tissues are exposed to the same reflux associated inflammatory triggers, the immune response observed in RE is clearly distinct from that in SQ of BE. The differences in immune responses in BE tissue might contribute to its susceptibility for transformation into intestinal metaplasia.

## Introduction

Barrett’s esophagus (BE) is an intestinal metaplasia in the esophagus and an important risk factor for development of EAC (esophageal adenocarcinoma). The molecular mechanisms underlying the pathogenesis of BE and the transition to EAC have been studied to some extent in recent years. Reflux Esophagitis (RE) is characterized by inflammation of the esophagus due to reflux of bile and acid and precedes development of BE [1]. Patients with erosive RE have a higher risk for development of BE compared to patients with non-erosive reflux esophagitis (NERD). It is still unclear why some patients with gastroesophageal inflammation develop Barrett’s esophagus and others do not [2], [3]. These findings implicate that the esophageal squamous epithelium of patients with BE is predisposed to develop metaplastic changes in response to reflux components. In patients who have undergone esophagectomy with esophago-gastric anastomosis, the development of intestinal metaplasia occurred significantly more often in patients who had BE preoperatively than those without BE. This was despite the presence of a similar degree of postoperative reflux esophagitis [4], [5].

It has been postulated that chronic inflammatory processes contribute to the enhanced sensitivity of RE/BE for transition into EAC [1], [6]–[9]. However, the immunological processes in these tissues have thus far not been studied in much detail with focus on differences in the amount and phenotype of T-cells in BE versus those in RE and EAC [10], [11]. Besides, the interpretation of these studies might be affected by a potential bias as the multilayered squamous epithelium in RE differs markedly from the single layered metaplastic epithelium in BE and the dysplastic EAC tissues. Yet, the studies by Fitzgerald *et al* are in favor for an aberrant inflammatory response in BE tissue, but direct analysis of the lymphocyte compartment is lacking in these studies. Therefore, it is important to describe in detail the phenotypes of lymphocytes found in both squamous epithelium and BE-epithelium in order to characterize the type of lymphocyte response. We have already provided evidence that the presence of lymphocytes in BE tissue reflects an altered tissue environment resembling duodenal tissue rather than the presence of inflammatory response. Good data regarding lymphocytes in not-affected squamous epithelium in the esophagus is lacking.

Few studies have been conducted to study squamous epithelium in Barrett’s esophagus patients and compare it to inflamed squamous epithelium of GERD patients and normal squamous esophageal epithelium in controls [1], [12]–[19]. Those studies revealed that the mRNA of caudal type homeobox 2 (CDX-2) protein was increased in squamous epithelium in GERD patients with BE compared to that of GERD patients without BE. As the CDX-2 protein serves a crucial role in embryogenesis and intestinal differentiation, this increase is believed to imply that the healthy looking esophageal squamous epithelium in BE patients might be primed towards transformation of intestinal metaplasia in the esophagus. This hypothesis was supported by a higher CDX2 expression in the squamous epithelium during development of BE in a mouse BE model [20]. Further support for a predisposed epithelium in BE comes from studies that observed clear differences in the MAP kinase pathway of BE versus GERD patients [15], [16]. All these data suggest that the healthy looking squamous epithelium is already primed towards metaplasia, probably as a consequence of an aberrant chronic inflammation driven microenvironment due to reflux of acid and bile. The purpose of this study was to analyse T-cells-phenotypes in squamous epithelium of Barrett’s esophagus patients, RE patients and controls, and compare these with those present in the inflamed RE tissue.

## Materials and Methods

### Patient characteristics and sample collection

50 patients were included in our study: 21 controls, 19 BE patients and 8 RE patients (see Table 1). In the 19 BE patients the diagnosis of BE was histologically confirmed by the presence of specialised intestinal metaplasia (IM) containing goblet cells in at least one of the biopsies. Only patients who had BE segments no less than C0M3 were included in the study to eliminate the possibility of sampling error while taking biopsies. RE patients were defined as having gastroesophageal reflux complaints and fulfilled endoscopic diagnostic criteria of Reflux Esophagitis according to Los Angeles classification [21]. Controls consisted of patients who visited our clinic for upper endoscopic examination for upper GI symptoms other than gastroesophageal reflux symptoms and had no history of gastroesophageal reflux disease (GERD). Symptoms were evaluated by a standardised questionnaire. BE patients, RE patients and controls were also excluded if they had any signs or history of immune-associated disorders and/or oncologic conditions.

**Table 1:** Patients and Controls characteristics.

	BE patients	Controls	RE patients
Number of patients	19	23	8 (7 grade A, 1 grade B and 1 grade C according to LA class)
Mean age (±SD)	62±13	49±18	53±17
Gender (% males)	79%	35%	63%
Presence of low grade dysplasia	16%	0%	0%
PPI use	95%	17%	25%
Hiatal Hernia	74%	22%	25%

Biopsies from BE patients were obtained of the macroscopically non-inflamed (normal looking) squamous esophageal tissue located 5 cm above the BE segment (BE SQ 5 cm). In controls, esophageal biopsies were obtained at 2 cm (C SQ 2 cm) and 5 cm (C SQ 2 cm) of the normal looking esophageal epithelium above the gastroesophageal junction. In RE patients the biopsies were obtained from the inflamed esophageal tissue (RE) and normal looking esophageal tissue located 5 cm above the inflammation (RE SQ 5 cm).

**Figure 1:**
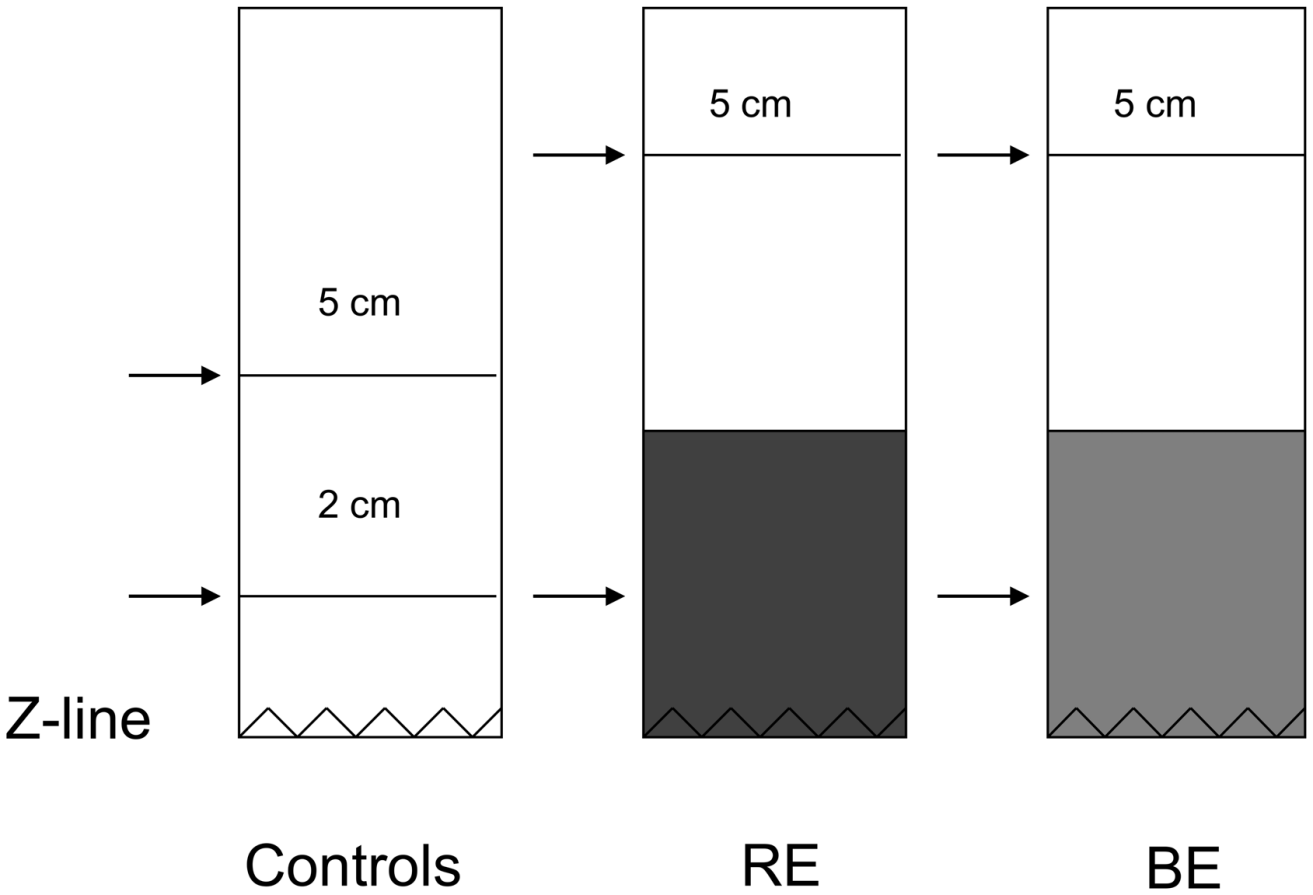
Illustration of the location of the taken biopsies. Arrows indicate the locations of the biopsying in controls, RE (Reflux Esophagitis) patients and BE (Barrett’s esophagus) patients.

As Reflux Esophagitis and BE are most prominent at the distal part of the esophagus, esophageal biopsies from normal healthy controls obtained from the distal part at 2 cm above the gastroesophageal junction, were compared with inflamed distal esophagus of patients with Reflux Esophagitis (Figure 1) [2], [22]. *Ex vivo* cultures of biopsies from proximal parts of the normal looking esophagus of all patient groups (BE, RE and controls) were compared with each other.

The study was approved by Medical Ethical Committee of the University Medical Center Utrecht and written informed consent was obtained from all patients and controls.

### *Ex-vivo* expansion of T-cells from small biopsies

Expansion of T-cells was performed according to a previously described method with small modifications [23]. Briefly, fresh biopsies were washed three times in IMDM medium (Lonza, Basel, Switzerland,) with 1.7 ug/mL amphotericin (Fungizone, Gibco, Invitrogen, Camarillo, CA, USA). Cellfoam matrices (9 mm×9 mm×1.5 mm; Cellsciences Pte Ltd, Singapore) were autoclaved, then incubated in 100 mg/ml rat tail collagen I (BD Biosciences, Bedford, MA) in phosphate-buffered saline (PBS) for 30 minutes at 37°C, followed by two rinses with PBS. Biopsies were placed on the matrix in a T-cell culture medium (IMDM, 14.2 M β-mercaptoethanol, penicillin, streptomycin, heat inactivated foetal calf serum (8%, Gibco), 1 mM Hepes, and 0.29 mg/ml freshly added L-glutamin (Gibco), with 10 units of IL-2 (BD Biosciences). Clark *et al*, previously showed that IL-2 alone already increased proliferation 5-fold while cells are preserving their homing phenotype [23]. Cells were harvested after 1, 2 and 3 weeks of culturing for staining and analysis by flowcytometry (FACS) (FACScalibur, Becton&Dickinson, Mountain View, CA, USA).

### Staining of cell surface markers by FACS

The FACS-based immunophenotyping of lymphocytes was carried out at day 14 of the *ex vivo* cultures. This was decided because a pilot study revealed that we needed two weeks of *ex vivo* expansion in order to have sufficient cells for FACS analysis (results not shown). Cells were washed with PBS supplemented with trisodium citrate (0.4% w/v, pH 7.4) and human pasteurised plasma solution (4 g/L; PBS2+) and subsequently incubated for 30 minutes on ice with directly labelled antibodies according to the instructions of the manufacturer. The following antibodies were used for the detection of specific cell surface markers: mAb CD3-FITC (clone sk7, 1∶20, BD Biosciences), CD3-PE (clone sk7, 1∶20, BD Biosciences), CD3-APC-Alexa 750 (clone UCHT1, 1∶25, Beckman Coulter), CD8-APC (clone SK1, 1∶100, BD Biosciences), CD8-PerCP (clone SK1, 1∶20, BD Biosciences), CD8-Pacific Blue (clone B9.11, 1∶20, Beckman Coulter, CD4-PerCP (clone L200, 1∶20, BD Pharmingen, San Diego, CA, USA), CD4-Krome Orange (clone 13B8.2, 1∶20, Beckman Coulter), CD103 (αE)-FITC, intraepithelial T-cell marker (Clone Ber-ACT8, 1∶20, BD Pharmingen), NKg2a (CD159a)-PE, inhibitory NK receptor, also present on cytotoxic T-cells (Clone Z199, 1∶50, Beckman Coulter). After washing with PBS2^+^, cells were resuspended in the same buffer and analysed by flowcytometry (depending on the analysis either a Gallios, Beckman Coulter, Woerden, The Netherlands or a Calibur, Beckman Dickinson, Mountain View, CA, USA).

### Intracellular granzyme B staining for FACS analysis

First, cells (around 10^5^ cells) were stained with the cell surface markers CD103 (αE)-FITC, Clone Ber-ACT8, BD Pharmingen) and CD8-PerCP (clone SK1, BD Biosciences). Then, cells were fixed in a fixation/permeabilization solution (eBioscience) for 10 minutes. After washing in a permeabilization solution (eBioscience), cells were incubated with mAb anti-granzyme B-PE (2 µg/ml, clone CLB-GB11, Sanquin, Amsterdam, The Netherlands) in the permeabilization solution (eBioscience) for 30 minutes. Cells were washed with PBS2+ and FACS analysis was performed.

### Statistical analyses

All statistical analyses were conducted using GraphPad Prism 5 (La Jolla, CA, USA). One-way analysis of variance with the non-parametric Kruskal-Wallis test was used to compare data from the proximal esophagus (three groups: C SQ 5 cm, RE SQ 5 cm and BE SQ 5 cm). The Dunn postmultiple comparison Test was performed to determine the significance between each group. Analysis of the data from the distal esophagus (two groups: C SQ 2 cm and RE) was conducted by Mann–Whitney U test. Data were expressed as mean±standard error of the mean (SEM). Analysis of paired groups (biopsies from different locations from the same patient) was conducted using paired t-test. A two-sided p-value <0.05 was considered to be statistically significant.

## Results

### Low numbers of CD3^+^CD4^+^-cells in normal looking squamous epithelium compared to RE tissue in *ex vivo* cultures

Lymphocytes from collagenated normal looking squamous esophageal biopsies contained less CD4^+^-cells in the CD3^+^-population (CD3^+^CD4^+^-cells) than the *ex vivo* expanded squamous epithelial biopsies (p = 0.003). This was due to some apoptosis of lymphocytes (data not shown).

**Figure 2:**
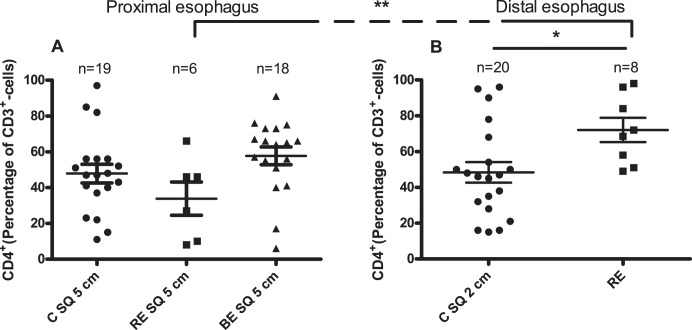
More CD3^+^CD4^+^-cells in *ex vivo* cultures of RE biopsies, compared to normal looking squamous esophageal tissue biopsies. Panel A depicts percentage of CD4^+^-cells of the population of CD3^+^-cells (CD3^+^CD4^+^) in 14 days *ex vivo* culture of normal looking squamous epithelial biopsies from controls (C SQ 5 cm: 5 cm above the Z-line), RE patients and (RE 5 cm: 5 cm above the inflamed RE tissue) and BE patients (BE SQ 5 cm: 5 cm above the BE tissue. Panel B represents CD3^+^CD4^+^-percentage in 14 days *ex vivo* culture of normal looking squamous epithelial biopsies of controls (C SQ 2 cm: 2 cm above the Z-line) and inflamed esophageal tissue of RE-patients (RE). In panel A Kruskal-Wallis test was used. In panel B, a Mann-Whitney test was used. Between panels A and B a paired t-test was used to compare CD3^+^CD4^+^-percentage in *ex vivo* cultures from RE tissue to normal looking squamous esophageal epithelium taken 5 cm above the gastroesophageal junction from the same RE patients (*p<0.05, **p<0.005). Mean value and SEM are calculated.

By immunophenotyping of the lymphocytes no differences in the percentage of CD3^+^CD4^+^-cells were observed between normal looking proximal squamous epithelium of Barrett patients, RE patients and controls (Figure 2, panel A). In contrast, in the inflamed esophageal tissue from RE patients a significantly higher percentage of CD3^+^CD4^+^-cells was observed (72±7% (mean ± SEM)) compared to visually non-inflamed distal esophageal tissue of controls (p = 0.01, Figure 2, panel B) and normal looking squamous esophageal tissue from RE patients (p = 0.007, Figure 2, panels A and B).

### Higher percentage of CD3^+^CD4^+^-cells is CD103^+^ in normal looking squamous esophageal compared to RE tissue

**Figure 3:**
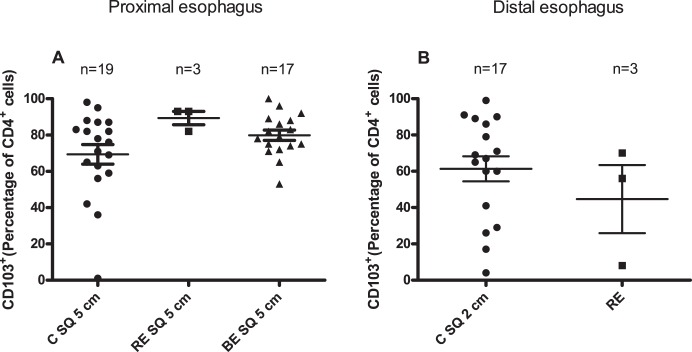
Normal looking squamous esophageal epithelium is characterized by higher percentage of CD4^+^CD103^+^-cells. Panel A depicts percentage of CD103^+^-cells of the population of CD4^+^-cells (CD4^+^CD103^+^) in 14 days *ex vivo* culture of normal looking squamous epithelial biopsies from controls (C SQ 5 cm: 5 cm above the Z-line), RE patients and (RE 5 cm: 5 cm above the inflamed RE tissue) and BE patients (BE SQ 5 cm: 5 cm above the BE tissue. Panel B represents CD4^+^CD103^+^-percentage in 14 days *ex vivo* culture of normal looking squamous epithelial biopsies of controls (C SQ 2 cm: 2 cm above the Z-line) and inflamed esophageal tissue of RE-patients (RE). Panel A: Kruskal-Wallis test. Panel B: Mann-Whitney test. Mean value and SEM are calculated.

CD103^+^ is a marker for tissue associated immunosuppressive lymphocytes [24]–[27]. No differences were found in the percentage of CD103^+^ inside the CD4^+^-cell population between squamous epithelium of Barrett patients and distal esophageal epithelium of controls (Figure 3, panel A). Also, the CD4^+^CD103^+^ percentage *in ex vivo* cultures of biopsies obtained from Reflux Esophagitis (RE) (45±19%) was not statistically different from *ex vivo* cultures of normal looking distal esophagus (C SQ 2 cm) (61±7%) (Figure 3, panel B). Although data for this staining were limited as only *ex vivo* cultures from 3 patients with Reflux Esophagitis were measured, significantly lower numbers of CD4^+^CD103^+^-cells were found in *ex vivo* cultures of RE when compared to normal looking proximal esophageal epithelium of BE patients (RE vs BE SQ 5 cm, p = 0.02) (Figure 3).

### Similar percentage of CD8^+^CD103^+^-cells and CD8^+^NKg2a^+^-cells in *ex vivo* cultures of inflamed (RE) and biopsies from normal looking esophagus

**Figure 4:**
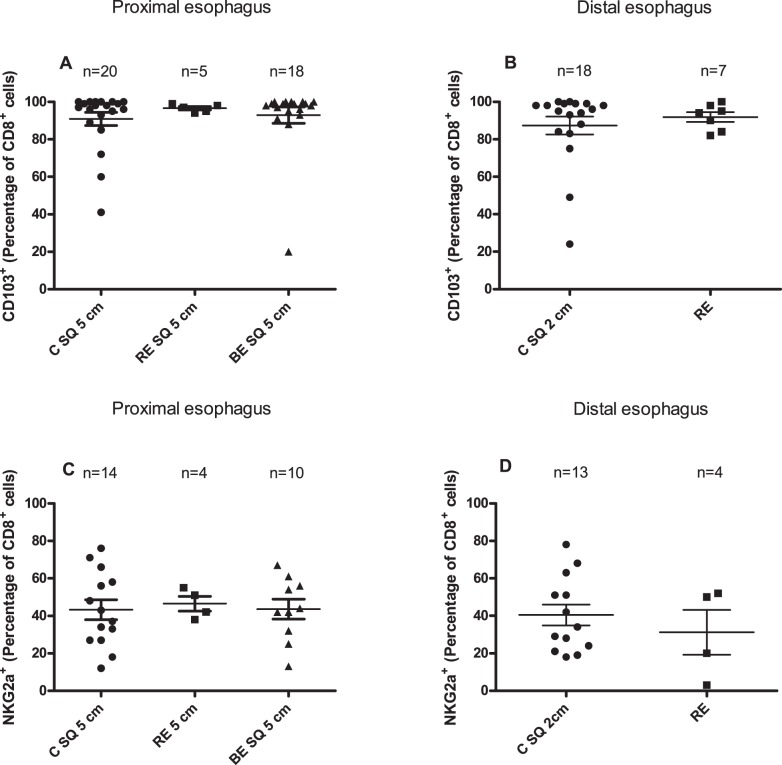
Similar percentage of CD8^+^CD103^+^ and CD8^+^NKg2a^+^-cells in RE and other squamous esophageal biopsies. Panel A depicts percentage of CD103^+^-cells percentage of the population of CD8^+^-cells (CD8^+^CD103^+^) in 14 days *ex vivo* culture of squamous epithelial biopsies from controls (C SQ 5 cm: 5 cm above the Z-line), RE patients and (RE 5 cm: 5 cm above the inflamed RE tissue) and BE patients (BE SQ 5 cm: 5 cm above the BE tissue). Panel B represents CD8^+^CD103^+^- percentage in 14 days *ex vivo* culture of normal looking distal squamous epithelial biopsies of controls (C SQ 2 cm: 2 cm above the Z-line) and inflamed esophageal tissue of RE-patients (RE). Panel C depicts percentage of NKg2a^+^-cells of the population of CD8^+^-cells (CD8^+^NKg2a^+^) in 14 days *ex vivo* culture of squamous epithelial biopsies from controls (C SQ 5 cm: 5 cm above the Z-line), RE patients and (RE 5 cm: 5 cm above the inflamed RE tissue) and BE patients (BE SQ 5 cm: 5 cm above the BE tissue. Panel D represents CD8^+^NKg2a^+^-percentage in 14 days *ex vivo* culture of squamous epithelial biopsies of controls (C SQ 2 cm: 2 cm above the Z-line) and inflamed esophageal tissue of RE-patients (RE). Panels A and C: Kruskal-Wallis test. Panels B and D: Mann-Whitney test. Mean value and SEM are calculated.

No differences were observed in the percentage of CD103^+^ cells in the CD8^+^-population (CD8^+^CD103^+^-cells) between the normal looking squamous epithelium from controls (C SQ 2 cm, C SQ 5 cm), BE patients (BE SQ 5 cm) and normal looking (RE SQ 5 cm) and inflamed epithelium from RE patients (RE) (Figure 4, panels A and B).

A similar percentage of NKg2a^+^-cells in the CD8^+^-population (CD8^+^NKg2a^+^-cells) was found in *ex vivo* cultures of squamous epithelial biopsies from normal looking esophagus of BE patients, RE patients and controls (Figure 4, panel C) and in *ex vivo* cultures of RE and distal esophagus of controls (Figure 4, panel D).

### High percentage of CD8^+^Granzyme B^+^-cells in RE

**Figure 5:**
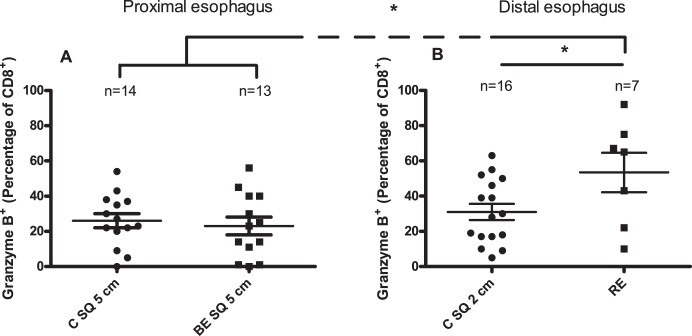
Higher percentage of CD8^+^GranzymeB^+^-cells in *ex vivo* biopsies from RE compared to *ex vivo* biopsies from normal looking squamous esophagus. Panel A depicts percentage of GranzymeB^+^-cells of the CD8^+^-population (CD8^+^Granzyme B^+^-cells) in 14 days *ex vivo* culture of squamous epithelial biopsies from controls (C SQ 5 cm: 5 cm above the Z-line) and BE patients (BE SQ 5 cm: 5 cm above the BE tissue. Panel B represents CD8^+^GranzymeB^+^-percentage in 14 days *ex vivo* culture of normal looking squamous epithelial biopsies of controls (C SQ 2 cm: 2 cm above the Z-line) and inflamed esophageal tissue of RE-patients (RE). Mann-Whitney test was used (*p<0.05). Mean value and SEM are calculated.

Granzyme B is a cytotoxic molecule present in cytotoxic CD8^+^-effector cells [28], [29]. No significant differences were found in the percentage of Granzyme B^+^ cells inside the CD8^+^-population (CD8^+^Granzyme B^+^-cells) between *ex vivo* cultures from normal looking epithelial biopsies from proximal esophagus: C SQ 5 cm, BE SQ 5 cm and C SQ 2 cm (Figure 5, panel A). A higher percentage of CD8^+^Granzyme B^+^-cells was found in *ex vivo* cultures of RE (53±11%) when compared to normal looking squamous epithelial tissue in distal and proximal esophagus (Figure 5, panels A and B). RE was significantly higher in CD8^+^Granzyme B^+^-cells compared with normal looking squamous esophageal tissue of controls (C SQ 5 cm) (26±4%, p = 0.04) and BE patients (BE SQ 5 cm) (23±5%, p = 0.04) (Figure 5, panels A and B).

### Percentages of CD103^+^-cells and CD103^–^cells of the CD8^+^GranzymeB^+^-population are higher in RE

**Figure 6:**
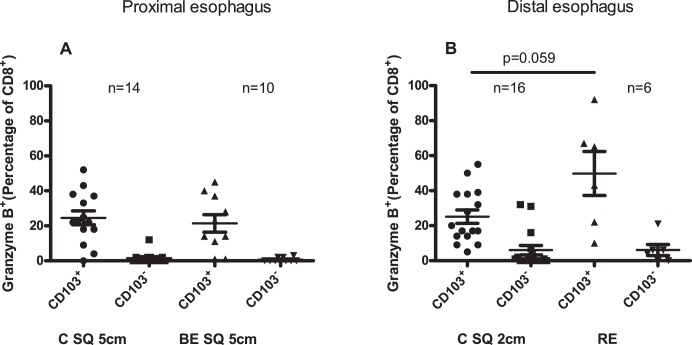
Higher percentage of CD103^+^cells and CD103^−^cells inside the CD8^+^GranzymeB^+^-population in RE. Panel A depicts percentage of CD103^+^-cells and CD103^–^cells inside the CD8^+^GranzymeB^+^-population in 14 days *ex vivo* culture of squamous epithelial biopsies from controls (C SQ 5 cm: 5 cm above the Z-line) and BE patients (BE SQ 5 cm: 5 cm above the BE tissue. Panel B represents CD103^+^ and CD103^–^cells inside the CD8^+^ GranzymeB^+^-population in 14 days *ex vivo* culture of normal looking squamous epithelial biopsies of controls (C SQ 2 cm: 2 cm above the Z-line) and inflamed esophageal tissue of RE-patients (RE). Mann-Whitney test was used. Mean value and SEM are calculated.

In the normal looking squamous esophagus of controls and BE patients, relatively low percentages of CD103^+^-cells inside the CD8^+^GranzymeB^+^-population (CD8^+^GranzymeB^+^CD103^+^) were found, 25±4% in proximal squamous esophageal epithelium of controls (C SQ 5 cm) and 21±5% in squamous esophageal epithelium of BE patients (BE SQ 5 cm) (Figure 6, panel A). That was lower, yet not significantly, compared to *ex vivo* cultures of RE tissue, where the highest percentage of CD8^+^GranzymeB^+^CD103^+^ (50±12.5%) was found (Figure 6, panel B). CD8^+^CD103^–^cells have a pronounced cytotoxic phenotype [24]. In normal looking proximal squamous esophageal epithelium very low percentages of CD103^−^cells inside CD8^+^GranzymeB^+^-population (CD8^+^GranzymeB^+^CD103^−^) were found (0.6±0.3% in BE SQ and 1.6±0.3% in C SQ 5 cm) (Figure 6, panel A). The percentage of CD8^+^GranzymeB^+^CD103^−^ was similar in inflamed tissue of RE (6±3%) and normal looking distal squamous esophagus from controls (C SQ 2 cm, 6±3%) (Figure 6, panel B). Compared to visually non-inflamed squamous esophageal tissue from BE patients (BE SQ 5 cm, 0.7±0.4%), RE had significant higher percentage of CD8^+^GranzymeB^+^CD103^–^cells (p = 0.048) (Figure 6, panels A and B).

### Percentage of CD8^+^GranzymeB^+^CD94^+^-cells is slightly higher in squamous epithelium of RE patients compared to normal looking squamous epithelium

**Figure 7:**
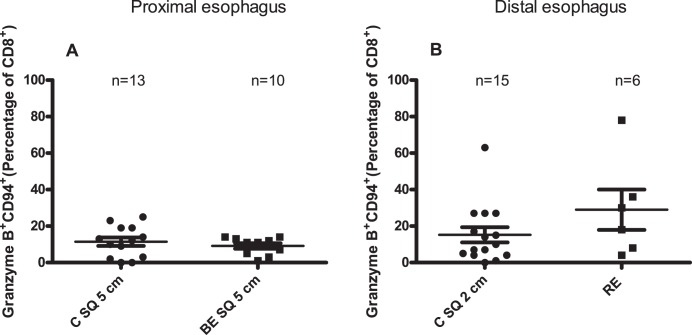
Higher percentage of CD8^+^GranzymeB^+^CD94^+^-cells in *ex vivo* cultures of RE compared to *ex vivo* cultures of proximal esophagus. Panel A depicts percentage of GranzymeB^+^CD94^+^-cells of the percentage of CD8^+^-cells (CD8^+^GranzymeB^+^CD94^+^) in 14 days *ex vivo* culture of normal looking proximal squamous epithelial biopsies from controls (C SQ 5 cm: 5 cm above the Z-line) and BE patients (BE SQ 5 cm: 5 cm above the BE tissue. Panel B represents CD8^+^ GranzymeB^+^CD94^+^ percentage in 14 days *ex vivo* culture of normal looking distal squamous epithelial biopsies of controls (C SQ 2 cm: 2 cm above the Z-line) and inflamed esophageal tissue of RE-patients (RE). Mean value and SEM are calculated.

NKg2a/CD94 is present on cells which are inhibited in cytotoxic degranulation [30]–[32]. We tested the expression of NKg2a/CD94 on Granzyme B^+^-cells. The percentage of CD8^+^GranzymeB^+^CD94^+^-cells was relatively low in normal looking proximal squamous esophageal epithelium of controls (C SQ 5 cm, 11±2%) and BE patients (BE SQ 5 cm, 9±1%) (Figure 7, panel A).

A higher percentage (yet not significant) of CD8^+^GranzymeB^+^CD94^+^-cells was found in RE (29±11%) (Figure 7, panel B).

## Discussion

Reflux Esophagitis is thought to be a mandatory first stage in the route towards the development of BE. Here we analysed the tissue-resident T-cell response of inflamed and normal looking squamous epithelium found in the esophagus of RE patients with the normal looking squamous esophageal tissue found proximal of the Barrett’s epithelium in BE patients and compared it with normal looking esophageal tissue from controls. We have chosen to exclude the intestinal type tissue found in BE from our analysis because the comparison of one layered cylinder epithelium of BE tissue with multiple layered squamous esophageal tissue is biased by comparing different tissue types.

In this study, it was our aim to investigate whether T-cell responses in normal looking squamous esophageal tissue from BE patients are similar to those found in inflamed esophageal tissue of RE patients, which might point at a predisposition for inflammation in the squamous esophageal tissue of BE patients.

There is not much known regarding the phenotype of tissue lymphocytes in normal squamous esophageal tissue let alone how these compare to those in patients with RE and BE. This is mainly due to the fact that for ethical reasons the research on the esophagus is limited to studies of small tissue biopsies that do not allow an in depth analysis of T-cell subsets. As a consequence, this research was either based on immunohistochemistry or PCR based analysis on whole tissue biopsies [1]; [18]; [31]; [32]. However, PCR does not provide data on individual cells and immunohistochemistry is often limited to maximum of 3 antigens, limiting extensive phenotyping of T-cells [10], [33], [34]. From the limited available data it was concluded that there is a number of tissue-resident CD8^+^-lymphocytes in healthy squamous esophagus whereas the total number of lymphocytes in Reflux Esophagitis was increased compared to non-inflamed esophagus [35], [36]. Other studies found that BE was associated with the presence of a CD4^+^ response, which is in line with our data [34], [37], [38]. Performing multicolour flowcytometric based immunophenotyping on *ex vivo* expanded T-lymphocytes not only allowed us to confirm these data but allowed a further characterisation of the tissue associated lymphocytes. The validity of this method has been described for skin biopsies, a tissue very reminiscent to squamous epithelium of the oesophagus [23].

It was shown, that PPI use can increase the number of intraepithelial lymphocytes in the colon [39]. As 17% of our controls and 25% of the RE patients were treated with PPIs, we studied a putative effect of PPI use in the control and RE groups on the composition of lymphocytes in *ex vivo* cultures from the esophageal tissue. No significant differences were found between controls using PPIs and those not using PPIs and between RE patients using PPIs or not (data not shown). It is tempting to speculate that the multilayered epithelium of the esophagus comprises a better barrier for the effect of PPI compared to the unilayered epithelium of the colon.

In RE, a higher percentage of CD3^+^CD4^+^-cells was found (Figure 2), compared to normal looking squamous esophageal epithelium of RE patients and distal esophageal epithelium of controls. To further define those cells we have applied a CD103 staining.

CD103 has been shown to be upregulated on intraepithelial lymphocytes and can be induced by TGF-β [40], [41] A higher expression of TGF-β was found in biopsies from healthy children compared to children with Reflux Esophagitis and Eosinophilic Esophagitis, which was in line with the hypothesis that the TGF-β-CD103 axis was disturbed under inflammatory conditions [33]. The low numbers of CD4^+^CD103^+^-lymphocytes in RE might indicate low levels of tissue TGF-β, which led to a diminished ability of CD4^+^-cells to upregulate CD103 or to an impaired influx of CD4^+^CD103^+^-cells from peripheral blood. Lower levels of TGF-β can point at a pro-inflammatory environment [42]. This is supported by the finding that CD103^+^-lymphocytes have an immunosuppressive phenotype placing them in an anti-inflammatory tissue environment [27], [43], [44]. Our results indicated that no significant differences in T-cell phenotypes were present between all macroscopically normal looking squamous esophageal tissues from controls, BE and RE patients. In addition, inflamed squamous epithelium of the esophagus of RE patients and normal looking tissue of the controls and BE patients had similarly high percentages of CD8^+^CD103^+^-cells (Figure 4). The presence of high percentage of CD8^+^CD103^+^-cells in normal looking esophageal tissue corresponded with high levels of TGF-β found in normal controls in a study with compared biopsies from patients with Eosinophilic Esophagitis and normals [33]. Interestingly, the source of TGF-β might be the CD103^+^CD8^+^-lymphocytes as these cells in the lung have been shown to produce TGF-β [24]. Due to the fact that TGF-β can be produced by many cell types, such as macrophages, intestinal epithelial cells, dendritic cells and regulatory T-cells, it is still unclear what the initial source of TGF-β is in esophageal tissue [24], [25], [41], [45]–[47].

CD103^+^CD8^+^-lymphocytes in the lung were found to be low in granzyme B, just as found by us in the esophagus (Figure 6 A) [24]. Piet el al have found that CD8^+^CD103^+^-cells could rapidly upregulate granzyme B after stimulation with IFN-γ [24], which might represent a mechanism to limit the danger of aspecific release of granzyme B from these cells under low inflammatory conditions. Indeed, these lung CD103^+^/CD8^+^ lymphocytes, had a higher mRNA expression of granzyme B, indicating the potency to quickly upregulate granzyme B in response to inflammatory/infectious conditions [24]. Whether a similar situation is present in the esophagus is yet to be determined.

A high percentage of CD8^+^GranzymeB^+^-cells was found in RE (Figure 5). This was in line with the hypothesis that a microenvironment was present in inflamed tissue of RE with high IFN-γ levels which in turn facilitated the CD8^+^CD103^+^ to become cytotoxic. Indeed, we found a high number of CD8^+^GranzymeB^+^CD103^+^ cells in RE and these lymphocytes from RE tissue produced significant amounts of IFN-γ *in vitro* (Figure S1). The analysis of the composition of Granzyme B^+^ cells showed that CD8^+^GranzymeB^+^CD103^+^ were over represented in RE compared to normal looking proximal squamous esophageal epithelium. Also, significantly more CD8^+^GranzymeB^+^CD103^–^cells were found in RE tissue compared to squamous esophageal epithelium of BE patients (Figure 6). These latter CD8^+^GranzymeB^+^CD103^–^cells in RE tissue might have been recruited directly from the circulation as their CD103 expression was still negative. This hypothesis was supported by the finding that normal looking squamous esophageal tissue had a low percentage of CD8^+^ Granzyme B^+^-cells and a relatively high percentage of CD8^+^ Granzyme B^+^ CD103^+^-cells.

Immunophenotyping of T-lymphocytes from *ex vivo* cultures from RE showed that RE was characterized by significantly higher percentage of CD3^+^CD4^+^-cells (Figure 2, panel B) and a significantly lower percentage of CD4^+^CD103^+^-cells compared to normal looking squamous epithelium from BE, RE and controls. However, conclusions should be drawn with caution as only 3 RE-patients were analyzed (Figure 3). Nevertheless, this finding may point at an increased presence of CD4^+^CD103^–^cells in RE tissue. As CD103 on epithelial lymphocytes is upregulated by TGF-β, this finding suggested that an increased homing of new lymphocytes (CD4^+^CD103^–^cells) was present from the circulation to RE, which in turn became CD103^+^ by tissue TGF-β [40]. Therefore, our findings are consistent with the hypothesis of an enhanced traffic of T-lymphocytes to RE tissue, compared to normal squamous esophageal epithelium. The percentage of CD8^+^CD103^+^-cells was high in RE which was similar to normal looking squamous esophageal epithelium (Figure 4) whereas the percentage of CD4^+^CD103^+^ was low in RE which was more corresponding with BE tissue (Figure 3) [38]. This may point at a discrepancy in TGF-β responses on CD4^+^ and CD8^+^ cells in RE and BE, which is in line with an aberrant TGF-β signalling in RE and BE already described before [33], [48].

The CD8^+^Granzyme B^−^CD103^−^ which were higher in RE compared to proximal normal looking squamous esophageal tissue might have been cells that were initially high in granzyme B but had downregulated Granzyme B by engagement of their inhibitory, NKg2a/CD94 complex. This complex was found higher on CD8^+^Granzyme B^+^-cells in RE. The NKG2a/CD94 complex is a heterodimer present on NK cells and CD8^+^-T-cells. A high expression of NKG2a and CD94 is associated with inhibition in TNF-production and degranulation of cytotoxic mediators [30], [31], [31], [32,49]. It is to speculate that NKg2a/CD94 prevents CD8^+^Granzyme B^+^ from obtaining CD103 on the surface. Alternatively, CD103 might not have been upregulated due to an aberrant TGF-β response (Figure 7) [24].

In conclusion, T-cells found in the tissue of RE point at an inflammatory T-cell response when compared with normal esophageal tissue in BE and RE patients and controls. Immune microenvironment from the normal looking squamous esophageal tissue from BE and RE patients was similar to that of controls. Our study has pointed out that normal looking esophageal tissue of BE, RE patients and controls has a completely different immunologic profile than inflamed tissue from RE patients, suggesting that processes other than inflammation are important in development of BE. New findings regarding similarities, differences and function of specific phenotypes of lymphocytes in BE, RE and control esophageal tissue can contribute to a better understanding regarding the role of inflammation in the development of BE from RE.

## Supporting Information

Figure S1 **IFN-γ production in T-lymphocytes after stimulation with PMA and ionomycin.** 56% of CD8^+^-cells were positive for IFN-γ after stimulation. There were no cells positive for IL4.
(TIF)
